# Serological Investigation on the Presence of Feline Coronavirus (FCoV) and Severe Acute Respiratory Syndrome Coronavirus 2 (SARS-CoV-2) in Domestic Cats Living with COVID-19 Positive Owners in the UAE, 2022

**DOI:** 10.3390/ani13030493

**Published:** 2023-01-31

**Authors:** Mohamed El-Tholoth, Mahmoud Hussein, Dina Mohammed, Majed Al-Rasheedi, Hamad Al-Qubaisi, Abdullah Al-Blooshi, Mohammed Al-Ahbabi, Zayed Al-Dhaheri, Khalifa Al-Blooshi, Majd Al-Herbawi, Eman A. Abo Elfadl, Rabiha Seboussi

**Affiliations:** 1 Veterinary Science Program, Faculty of Health Sciences, Al Ain Men’s Campus, Higher Colleges of Technology, Al Ain P.O. Box 17155, United Arab Emirates; 2 Department of Virology, Faculty of Veterinary Medicine, Mansoura University, Mansoura 35516, Egypt; 3 Animal Development & Health Department, Ministry of Climate Change and Environment (MOCCAE), Dubai P.O. Box 1509, United Arab Emirates; 4 Department of Animal Husbandry and Development of Animal Wealth, Faculty of Veterinary Medicine, Mansoura University, Mansoura 35516, Egypt

**Keywords:** cats, feline coronavirus, SARS-CoV-2, ELISA, UAE

## Abstract

**Simple Summary:**

The current study represents the first serological investigation on the presence of FCoV and SARS-CoV-2 in domestic cats in the UAE. A total of 83 sera were obtained from domestic cats living with COVID-19-positive owners (by RT-qPCR). Seroprevalence of FCoV in collected samples from all cats was 65% (54/83). All cat breeds could be infected with FCoV. Purebred cats showed a higher seropositivity rate than mixed-breed cats. British short-hair breed showed the highest prevalence rate, while Siamese showed the lowest rate. Cats that have outdoor access are less susceptible to becoming infected than those without outdoor access. Antibodies to SARS-CoV-2 were detected in cats’ population at a low prevalence, eight (9.6%) cats were considered positive. Both FCoV and SARS-CoV-2 antibodies were detected in four samples (4.8%). COVID-19-positive patients should avoid close contact with their cats during illness time.

**Abstract:**

Feline coronavirus (FCoV) is widely circulating among domestic cats (*Felis catus*). The zoonotic origin of the emerged severe acute respiratory syndrome coronavirus-2 (SARS-CoV-2) and the biological characteristics of CoVs, including the ability to cross interspecies barriers, facilitate its emergence in different animals, including cats’ populations. The current study is the first to report the serological investigation on the presence of FCoV and SARS-CoV-2 in domestic cats living with COVID-19-positive owners in the UAE. A total of 83 sera were collected from domestic cats living with COVID-19-positive owners (by RT-qPCR). The cats were sampled during the period between February and May 2022 in Al-Ain and Abu Dhabi Cities, UAE. Detection of FCoV and SARS-CoV-2 was carried out by enzyme-linked immunosorbent assay (ELISA). FCoV antibodies were detected in 54 samples (65%). The frequencies of FCoV were significantly higher in purebred cats (48%; 40/83) and in the cat group with outdoor access (49.4%; 41/83). SARS-CoV-2 seroprevalence in collected sera revealed 8 samples (9.6%) with positive results. Four samples (4.8%) showed positive results for both FCoV and SARS-CoV-2 antibodies. In conclusion, FCoV is widely circulating within cats’ populations involved in the study. The antibodies for SARS-CoV-2 were detected in cats’ populations but at a low prevalence rate. COVID-19-positive people should avoid close contact with their cats. Future serological testing of large cats’ populations is crucial for providing a good understanding of COVID-19 dynamics in cats.

## 1. Introduction

Feline coronavirus (FCoV) is a major worldwide infection in cats’ populations. FCoV is an enveloped RNA virus that is classified within the order *Nidovirales*, family *Coronaviridae*, and genus Alphacoronavirus. There are two FCoV serotypes: Type I and type II. The virus has two biotypes, Feline Enteric Coronavirus (FECV) and the feline infectious peritonitis virus (FIPV) [1]. FECV is commonly circulating among cats, especially in those housed at high density or in groups, while FIP usually develops sporadically [2]. A fundamental event for the FIP development is a series of FCoV nucleic acid mutations responsible for switching the cell tropism of the virus from enterocytes for FECV to monocytes/macrophages for FIPV [1,2]. FCoV transmission occurs by the oral-fecal route [3]. Cats infected with FCoV stay asymptomatic, about 1–12% of infected cats progress to FIP [4,5,6,7]. FIP is characterized by an extremely high mortality rate [8]. The clinical signs are categorized into signs with effusions (wet form) and those associated with granuloma formation at localized sites in the body (dry form). In some cases, both wet and dry manifestations may occur in infected cats [3].

Prevention of FIP development relies on the prevention of FCoV infection in cats that have not yet been infected with FCoV. As per the FCoV antibodies screening on cats’ admission, it is possible to segregate seronegative cats away from positive ones [9]. There are several methods to detect FCoV antibodies. For shelter purposes, the technique should be reliable, fast, and inexpensive [10].

SARS-CoV-2, the causative agent of the COVID-19 pandemic, causes high illness rates. SARS-CoV-2 is a member of the genus *Betacoronavirus* within the family *Coronaviridae* [11]. In the UAE, the first COVID-19 case was identified on 29 January 2020 [12]. To combat this pandemic, the UAE has taken different steps to ensure the safety of its citizens and residents, including the adoption of a national project for mass screening and testing by polymerase chain reaction (PCR) for early identification of positive cases and subsequently implementing suitable control measures [13,14]. As reported to WHO till 23 December 2022, a total of 1,046,359 cases have been confirmed to be COVID-19 in the UAE (https://covid19.who.int/region/emro/country/ae, accessed on 3 January 2023).

Carnivores, such as cats, ferrets, minks, and to a lesser extent, dogs, were found to be susceptible to both experimental and natural infection with SARS-CoV-2 [15,16,17]. Some SARS-CoV-2-infected cats revealed respiratory and digestive disturbances [18,19]. Human-to-animal transmission of SARS-CoV-2 poses a serious risk due to the possibility of a more transmissible and pathogenic strain emerging. The susceptibility of animals to SARS-CoV-2 depends mainly on the structure of the angiotensin-converting enzyme 2 (ACE2), which is the virus receptor on the host cell. The feline ACE2 is one of the most closely related to human ACE2 [20]. Domestic cats commonly live in close contact with their owners, as well as with other animals when they go out, therefore, cats deserve special attention.

Serological investigations have revealed a variable prevalence of COVID-19 infection in cats, with a relatively low prevalence in comparison to the human population, as reviewed by [21]. The highest reported seroprevalences were reported in Peru (31.7%) and China (10.8%) when samples were collected from cats with owners who had a COVID-19 history [22,23].

As per our knowledge, the present study reports the first serological investigation on the presence of FCoV and SARS-CoV-2 in domestic cats living with COVID-19-positive owners in the UAE.

## 2. Materials and Methods

### 2.1. Ethical Statement

Our research work involved the use of non-experimental animals. All experiments were conducted in harmony with The Animal Research: Reporting of In Vivo Experiments (ARRIVE) and approved by the Veterinary Sciences Program, Faculty of Health Sciences, Al Ain Men’s Campus, Higher Colleges of Technology, Al Ain, UAE; and Animal Development and Health Department, Ministry of Climate Change and Environment (MOCCAE), UAE.

### 2.2. Animals and Sampling

**Figure 1:**
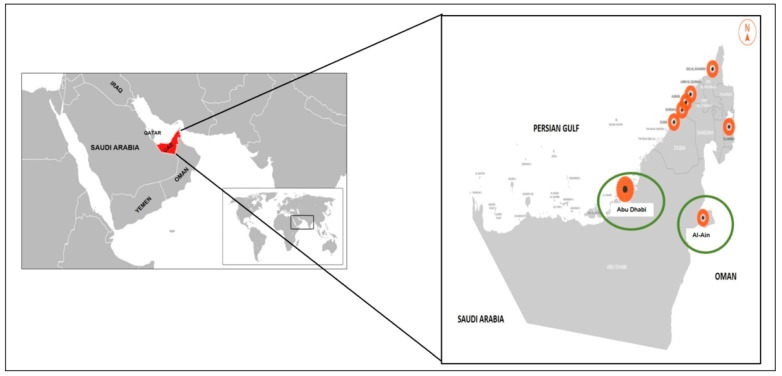
United Arab of Emirates (UAE) map showing Al-Ain and Abu-Dhabi cities (in green circles) involved in this study.

Our study used a nonprobability (convenience) sampling method. A total of valid 83 sera were collected from household cats living with COVID-19-positive owners (by RT-qPCR). The cats were sampled during the period between February and May 2022 in Al-Ain and Abu Dhabi cities, UAE (Figure 1). Cats aged less than 6 months were excluded from our study to avoid possible detection of maternal antibodies against SARS-CoV-2 and/or FCoV in young cats. Blood samples were withdrawn aseptically from cephalic or jugular veins (0.5 to 1 mL) and kept in a siliconized glass tube containing clot activator gel (Vacutainer^®^, Becton Dickinson, Plymouth, United Kingdom) to separate serum. The samples were centrifuged for 10 min at 4000× *g*, and the sera were kept in microtubules (1.5 mL) free of nucleases. The collected sera were stored at −20 °C until use. Heat inactivation of collected sera was conducted by incubation for 30 min at 56 °C before use. Sample collection was conducted with proper personal protective equipment, including gloves, head covers, N95 masks, goggles, and disposable gowns. Information regarding the age, sex, breeds, rearing model (solitary or in groups), reproductive status (whole, castrated, or ovariectomized), and access to the outdoors was recorded (Table 1).

### 2.3. Detection of Feline Coronavirus Antibodies in Cats’ Sera

Serum samples were tested for antibodies against FCoV using commercial indirect ELISA. Total Ab Test, Ingezim Corona Felino, Indirect ELISA for the detection of antibodies against FCoV in Cat serum (Ingenasa, Madrid, Spain) was used according to manufacture instructions. Briefly, the plate was already coated with the FCoV-specific antigens. Two hundred µL of 1/200 sera dilutions were added to the plate and kept at 37 °C for one hour. The plate was washed, and the specific peroxidase-labeled conjugate was added, followed by plate incubation for 1 h at 37 °C. The second washing of the plate was conducted and followed by the addition of the substrate to the wells. The stop solution (0.5 M H_2_SO_4_) was added to the wells after 20 min. The optical densities (OD) were measured at 450 nm using a Microplate Reader Model 680 (BIO-RAD, Hercules, CA, USA). The cut-off value was calculated based on the kit manual. OD value of samples higher than the cut-off was marked as positive. OD value lower than the cut-off was marked as negative. Negative and positive samples were involved in each experiment.

### 2.4. Detection of SARS-CoV-2 Antibodies in Cats’ Sera

Double antigen, multispecies ELISA based on the nucleocapsid antigen (ID Screen^®^ SARS-CoV-2 Double Antigen Multi-species, IDVet, Grabels, France) was used as per the instructions of the manufacturer. The assay had no cross-reactivity with antibodies specific for other coronaviruses or other agents related to human respiratory illnesses. The assay had a 100% sensitivity and specificity [24]. Briefly, 25 µL serum samples were added to the plate that was already coated with the purified recombinant N protein. The plate was incubated for 1 hr at 37 °C, then washed thrice. To bind to the anti-SARS-CoV-2 antibodies free Fab, a purified N-protein recombinant antigen horseradish peroxidase (HRP) conjugate (100 µL) was added and incubated for 1 h at 37 °C before being washed three times. The substrate, 3,3′,5,5′-tetramethylbenzidine (TMB) was used, and the reaction was stopped by 0.5 M H_2_SO_4_ after 20 min.

Microplate Reader Model 680 (BIO-RAD, Hercules, CA, USA) measured the optical densities (OD) at 450 nm. The cut-off value was calculated based on the kit manual. Samples with OD values higher than the cut-off were considered positive. Samples with OD values lower than the cut-off were considered negative. Negative and positive controls were involved in every run.

### 2.5. Statistical Analysis

Data were summarized, organized, and analyzed by SPSS version 23 (SPSS Inc., Chicago, IL, USA). Chi-square test (χ^2^) was used to compare the frequencies of ELISA results within different predictors. Then, the binary logistic regression analysis model was conducted to check the significance of different determinants to prediction risk factors for infection where ELISA test result (+ve or −ve) was used as dependent variables whereas location, age, breed, gender, reproductive status, rearing model and outdoor access as independent variables. The LR model was as follows:

Logit (P) = Ln (P1 − P) = βo + β1xi1 + β2xi2 + … + βkxik(1)

The term p/(1 − p) is the odds ratio; βj is the value of the jth coefficient, j = 1, 2, 3…, k and xij is the value of the ith. Case of the jth independent variable. The parameters of BLR are βo, β1 …, βk.

By taking the exponential function for the previous equation, the probability of occurrence of a condition can be estimated using the following logistic regression model:

P (Yi = 1 | Xi) =TiT Ti iβ Xβ Xβ Xodds e 11 odds 1 (e) 1 e−= = + + + (2)

where Yi is the binary outcome, Xi is the independent variable. The base e is the exponential function [10]. *p*-Value ≤ 0.05 was the statistical significance level. Odds ratios (ORs) were calculated with corresponding 95% confidence intervals (95% Cis) via a logistic regression model employing the Wald test.

## 3. Results

### 3.1. Detection of Feline Coronavirus Antibodies in Cats’ Sera

**Figure 2:**
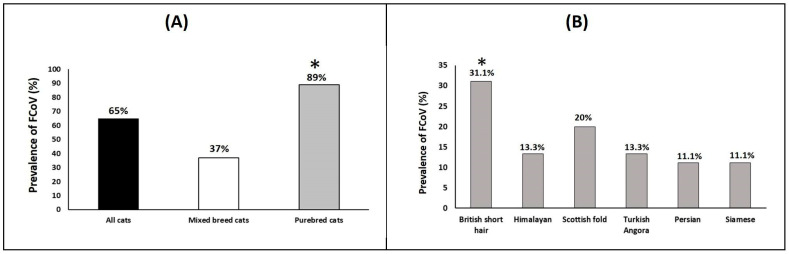
(**A**) Prevalence of feline coronavirus (FCoV) in cats (all cats, mixed-breed cats, and purebred cats). (**B**) Prevalence of feline coronavirus (FCoV) in different purebred cats (British short hair, Himalayan, Scottish fold, Turkish Angora, Persian, and Siamese). * statistically significant difference (*p* ≤ 0.05).

A total of 83 sera from 7 breeds (British short hair, Himalayan, Scottish fold, Turkish Angora, Persian, Siamese, and mixed-breed) of cats were used in our study. The seroprevalence of FCoV among collected samples from all cats was 65% (54/83). Seroprevalences were 63% (39/62) and 71% (15/21) in cats from AL-Ain and Abu Dhabi cities, respectively. FCoV antibody detection rate among purebred cats (89%; 40/45) was greater than mixed-breed cats (37%; 14/38). Among purebred cats, British short-hair breed showed the highest prevalence rate (31.1%; 14/45), while both Siamese and Persian cats showed the lowest rate (11.1%; 5/45) (Figure 2A,B). Table 2 shows the only variables that showed statistical significance are breed and access to the outdoors. Purebred cats had a higher rate of infection risk than mixed-breed cats (*p*-value ≤ 0.0001, OR = 16.96, 95% CI = 4.74–58.76). Moreover, FCoV infection among cats without outdoor access was greater than those with outdoor access (*p*-value = 0.02, OR = 0.211, 95% CI = 0.547 −3.27). The geographic location (Al-Ain city or Abu Dhabi city), rearing model, reproductive status, and gender were not significantly associated with FCoV antibodies in this population, although the rate of antibody detection was higher in Abu Dhabi city, among female cats, and in cats less than 1-year of age.

### 3.2. Detection of SARS-CoV-2 in Cats’ Sera

After analysis of 83 collected cats’ sera for antibodies to SARS-CoV-2, eight (9.6%) cats were considered positive for SARS-CoV-2 by ELISA (Table 3). Four samples (4.8%) tested positive for both FCoV and SARS-CoV-2 antibodies. No statistical significance was recorded for any of the variables. The eight affected cats are females, without outdoor access, and non-castrated. The antibody detection rate was higher (62.5%; 5 out of 8) in Al-Ain city, in purebred cats, cats older than 1 year, and those who were reared solitarily.

## 4. Discussion

Coronaviruses (CoVs) have long been known to cause infection in both animals and humans. FCoV infection is widely circulated in cats [6,25]. There have been reports revealing that felids, whether domesticated or wild, have been shown to be highly susceptible to experimental as well as natural infections with SARS-CoV-2 [26,27,28,29]. Household cats live in close contact with their owners as well as other animals when they go out, so cats could play a role in the evolution of SARS-CoV-2. As per our knowledge, the present study reports the first serological investigation on the presence of FCoV and SARS-CoV-2 in domestic cats living with COVID-19-positive owners in the UAE.

Our study showed that FCoV infection is widely distributed in the used cat populations in Al-Ain and Abu Dhabi cities, with a high seropositivity of 65%. Seroprevalence in the other studies showed varying results [4,30,31,32,33,34,35]. The findings revealed that FCoV was widely distributed in the cat populations studied. For example, our seroprevalence rate is higher than those reported previously, particularly in Australia (34%) [31], Italy (39%) [36], and Turkey (37%) [37], whereas the molecular prevalence was 21.5% and 31.8% in Italy, and Germany, respectively [38,39]. These varying results could be attributed to the sampling method, the examined cats’ population, the sample size, the geographic locations, and the test methods used.

Our results showed that all cat breeds can become FCoV-infected. Purebred cats revealed higher seropositivity than mixed-breed cats [40,41]. The high prevalence rate in these purebred cats could be the result of hereditary risk factors concentration caused by inbreeding [30]. Among the purebred cats, British short-hair breed showed the highest prevalence rate, while Siamese showed the lowest rate. These results are in harmony with the previous studies that reported that some of the breeds could be more susceptible to infection than others [30,40,42,43,44].

Among the risk factors studied in our study, only breed and outdoor access showed statistical significance. Cats that have outdoor access are less likely to be infected than those without outdoor access. The confined places are associated with increased exposure to a great concentration of FCoV, excreted mainly through feces [44]. Although other risk factors did not show statistical significance, female cats, and those less than one year old showed higher seropositivity. There is no clear biological explanation for the susceptibility and resistance of genders to FCoV, and the varying results among studies could be related to FCoV exposure and lifestyles of males and females [37]. FCoV could infect cats of all ages. Young cats (less than one year) and cats older than 10 years (not included in our study) are considered high-risk cats due to their relatively weak immunity [4,44,45].

**Figure 3:**
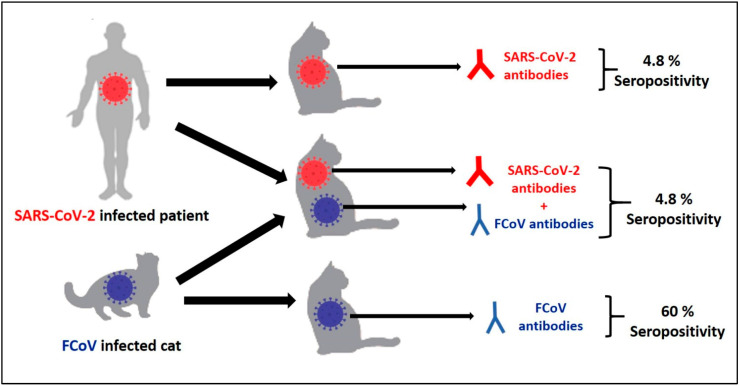
Seropositivity of cats’ population (*n* = 83) for feline coronavirus (FCoV) alone (60%), SARS-CoV-2 alone (4.8%) and both FCoV and SARS-CoV-2 together (4.8%).

To the best of our knowledge in the UAE, there are no published data on the seroprevalence of SARS-CoV-2 in cats. In the present study, the seroprevalence of SARS-CoV-2 in collected sera from the UAE revealed that eight samples out of 83 sera (9.6%) showed positive results. Four samples (4.8%) were positive for both FCoV and SARS-CoV-2 antibodies (Figure 3). Previous seroepidemiological studies revealed that cats can become infected with SARS-CoV-2 through their COVID-19-positive owners. SARS-CoV-2 infections in cats have been documented in different countries, e.g., in Hong Kong, Turkey, Belgium, USA, France, China, Spain, Germany, and UK [19,23,29,46,47,48,49,50].

These results suggest that cats in the UAE could be infected with SARS-CoV-2. It is important that COVID-19-positive cat owners be aware of this to avoid transmission of SARS-CoV-2 to their cats. The preventive measurements should be implemented based on a “One Health” approach. Further studies on bigger cats’ population are crucial. The study proposed the possibility of mixed infection, which could result in the recombination of FCoV and SARS-CoV-2, potentially affecting the pathogenicity and dynamics of both viruses.

## 5. Conclusions

In conclusion, FCoV is widely circulating within cats’ populations in in Al-Ain and Abu Dhabi Cities, UAE 2022. The Seroprevalence of FCoV from all cats was 65% (54/83). All cats’ breeds could become infected with FCoV. Purebred cats revealed higher seropositivity than mixed-breed cats. British short-hair breed showed the highest prevalence rate. Cats with access to the outdoors are less likely to become infected than those who do not. The other risk factors (locations, age, gender, reproductive status, and rearing model) did not show statistical significance. SARS-CoV-2 antibodies were detected in cats’ populations at low prevalence. COVID-19-positive patients should avoid close contact with their pets during the time of their illness. The preventive measures should be implemented based on a “One Health” approach. The study proposed the possibility of cats‘ co-infection with FCoV and SARS-CoV-2. Future serological testing of large cats’ population is crucial for providing a good understanding of COVID-19 dynamics in cats.

## Figures and Tables

**Table 1:** Details of collected samples from cats’ populations.

Regions	No. of Samples	Gender		Age Range		Breed		Access to the Outdoors		Reproductive Statues		Rearing Model	
		Male	Female	<1 year	≥1 year	Purebred	Mixed breed	Yes	No	Whole	Castrated or Spayed	Solitary	In Groups
Al-Ain	62	28	34	35	27	36	26	19	43	62	0	44	18
Abu-Dhabi	21	12	9	12	9	9	12	8	13	19	2	18	3
Total	83	40	43	47	36	45	38	27	56	81	2	62	21

**Table 2:** Prevalence of FCoV infection and analysis of risk factors in cats (*n* = 83).

Variables	FCoV Antibody Detection Rate		χ^2^	*p*-Value ^a^	ORs	95% CIs
	Numbers (Infected/Total)	%				
1—Geographic locations						
Al-Ain city	39/62	63	0.501	0.479	0.329	0.128–1.20
Abu Dhabi city	15/21	71				
2—Age						
<1 year	32/47	68	0.436	0.509	1.18	0.382–3.66
≥1 year	22/36	61				
3—Breed						
Purebred cats	40/45	89	24.55	<0.0001 ^a^	16.96	4.74–58.76
Mixed-breed cats	14/38	37				
4—Gender						
Male	25/40	62.5	0.223	0.637	0.402	0.130–1.24
Female	29/43	67.4				
5—Reproductive status						
Castrated or spayed	2/2	100	*	*	*	*
Whole	52/81	64				
6—Rearing model						
Solitary	39/62	63	0.502	0.479	0.562	0.191–1.56
Group	15/21	71				
7—Outdoor access						
Yes	13/27	48	5.03	0.02 ^a^	0.211	0.547−3.27
No	41/56	73				

^a^ Significant *p*-value ≤ 0.05. * Insufficient number of cats to allow statistical calculations.

**Table 3:** Details of the eight cats’ sera showed SARS-CoV-2 positive results.

Sample No.	Geographic Location	Breed	Gender	Age	Rearing Model	Outdoor Access	Reproductive Status
10	Abu Dhabi	Himalayan	Female	≥1 year	Group	No	Whole
27	Abu Dhabi	Mixed-breed	Female	≥1 year	Group	No	Whole
28	Abu Dhabi	Mixed-breed	Female	≥1 year	Group	No	Whole
40	Al-Ain	Mixed-breed	Female	<1 year	Solitary	No	Whole
67	Al-Ain	British short-hair	Female	<1 year	Solitary	No	Whole
75	Al-Ain	British short-hair	Female	≥1 year	Solitary	No	Whole
76	Al-Ain	Scottish fold	Female	<1 year	Solitary	No	Whole
79	Al-Ain	Persian	Female	≥1 year	Solitary	No	Whole

## Data Availability

The data that support the findings of this study are available from the corresponding author upon reasonable request.
